# Benign breast disease, recent alcohol consumption, and risk of breast cancer: a nested case–control study

**DOI:** 10.1186/bcr1039

**Published:** 2005-05-16

**Authors:** Rulla M Tamimi, Celia Byrne, Heather J Baer, Bernie Rosner, Stuart J Schnitt, James L Connolly, Graham A Colditz

**Affiliations:** 1 Channing Laboratory, Department of Medicine, Brigham and Women's Hospital and Harvard Medical School, Boston, MA, USA; 2 Department of Epidemiology, Harvard School of Public Health, Boston, MA, USA; 3 Cancer Genetics and Epidemiology Program, Lombardi Comprehensive Cancer Center, Georgetown University, Washington, DC, USA; 4 Department of Biostatistics, Harvard School of Public Health, Boston, MA, USA; 5 Department of Pathology, Beth Israel Deaconess Medical Center and Harvard Medical School, Boston, MA, USA; 6 Harvard Center for Cancer Prevention, Boston, MA, USA

## Abstract

**Introduction:**

Alcohol consumption is a well-established risk factor for breast cancer. Some studies have suggested that the risk of breast cancer associated with alcohol consumption is greater for women with a history of benign breast disease (BBD). We hypothesized that among women with biopsy-confirmed BBD, recent alcohol consumption would increase the risk of breast cancer in women with proliferative breast disease to a greater extent than in women with nonproliferative breast disease.

**Methods:**

We conducted a nested case–control study in the Nurses' Health Study I and II. The cases (*n *= 282) were women diagnosed with incident breast cancer, with a prior biopsy-confirmed breast disease. The controls (*n *= 1,223) were participants with a previous BBD biopsy, but without a diagnosis of breast cancer. Pathologists reviewed benign breast biopsy slides in a blinded fashion and classified the BBD as nonproliferative, proliferative without atypia, or atypical hyperplasia, according to standard criteria.

**Results:**

Women with nonproliferative breast disease consuming ≥ 15 g of alcohol per day had a nonsignificant 67% increased risk of breast cancer (odds ratio = 1.67; 95% confidence interval 0.65 to 4.34) compared with nondrinkers. There was no evidence that recent alcohol consumption increased the risk of breast cancer to a greater extent in women with proliferative BBD than among women with nonproliferative BBD (*P *for interactio *n *= 0.20).

**Conclusion:**

Contrary to our *a priori *hypothesis, there was no evidence that recent alcohol consumption increased the risk of breast cancer to a greater extent among women with proliferative BBD than among women with nonproliferative BBD.

## Introduction

Alcohol consumption is a well-established risk factor for breast cancer. Among those who drink, recent alcohol consumption of two or more drinks a day is associated with an approximately 30% increased risk of breast cancer compared with nondrinkers [1-3]. A pooled analysis of over 32,000 women participating in six prospective cohort studies [1], and at least three meta-analyses [2,4,5], confirmed that risk of breast cancer increases monotonically with increasing alcohol consumption [1,2].

Data from epidemiologic studies also suggest that alcohol may influence early stages as well as later stages of breast carcinogenesis. The primary evidence that alcohol may have an effect on early stages in the carcinogenic process comes largely from studies of alcohol consumption and mammographic density. Breast density is a strong predictor of breast cancer risk and is considered to be an early biomarker of breast cancer [6-9]. In most studies, recent alcohol consumption is positively associated with mammographic density [10-12]. Recent alcohol consumption has not been associated with increased incidence of proliferative benign breast disease (BBD) [13-15], although alcohol consumption between the ages of 18 and 22 has been associated with an increased risk of proliferative breast disease in one study [13].

A few studies have suggested that the increased risk of breast cancer associated with alcohol consumption is greater for women with a history of BBD [16,17], although the interactions were not statistically significant and this relation has not been observed in all studies [18]. BBD comprises multiple histologic subtypes. In prospective studies, where investigators have examined the risk associated with histologic subtypes of BBD, the proliferative lesions and in particular those with atypia were associated with the highest risk [19-21]. We hypothesized that among women with biopsy-confirmed BBD, alcohol consumption would increase the risk of breast cancer in those with proliferative breast disease to a greater extent than in women with nonproliferative breast disease.

## Materials and methods

### Study population

The Nurses' Health Study (NHS I) cohort was initiated in 1976, when 121,700 US registered nurses ages 30 to 55 returned an initial questionnaire. The Nurses' Health Study II (NHS II) is also an ongoing cohort study of over 116,000 US female nurses who were 25 to 42 years of age in 1989 when the study was initiated. Every 2 years, information on reproductive variables, body mass index, exogenous hormone use, and disease outcomes is collected in both cohorts. Semiquantitative food frequency questionnaires were included as part of the biennial questionnaire in 1980, 1984, 1986, and 1990 in NHS I, and in 1991 in NHS II. Alcohol consumption was also assessed on the baseline 1989 questionnaire in NHS II. The methods developed to follow up participants and confirm incident cancers and death in the Nurses' Health Study have been described previously in detail elsewhere [22] and have been applied to NHS II.

### Benign breast disease

Beginning with the initial NHS I questionnaire in 1976, participants have been asked on every biennial questionnaire to report any diagnosis of fibrocystic disease or other BBD. Early questionnaires (1976, 1978, and 1980) asked whether the respondent had ever been diagnosed as having 'fibrocystic disease' or 'other BBD' and whether she had been hospitalized with this diagnosis. Beginning in 1982, the NHS I questionnaires sought specific details of a history of biopsy-confirmed BBD. The initial 1989 NHS II questionnaire and all subsequent biennial questionnaires also asked participants to report any diagnosis of BBD and to indicate if it was confirmed by biopsy or aspiration.

### Selection of breast cancer cases and controls

We conducted a case–control study nested within the subcohort of women with a biopsy-confirmed BBD. Incident breast cancer cases in both cohorts were identified through the nurses' own reports and were confirmed by review of medical records. Cases in the study are cases of breast cancer diagnosed by 1 June 1994 (NHS I) or 1 June 1995 (NHS II) with a previous confirmed BBD biopsy and available pathology specimens (histologic sections and/or tissue blocks). Controls are women who did not have a diagnosis of breast cancer when the case was diagnosed and also had a previous biopsy-confirmed BBD and available pathology specimens. Controls were matched to cases on year of birth and year of biopsy. Attempts were made to identify four matched controls for each case, although this was not always possible. This study was approved by the Committee on Human Subjects at Brigham and Women's Hospital.

We identified incident confirmed breast cancer cases diagnosed after the return of the initial questionnaire through the 1994/1995 follow-up cycle and controls that also reported a previous biopsy-confirmed BBD. A total of 1,080 cases were originally identified for this study, and 4,353 controls matched on age and year of benign breast biopsy were selected. After selection, 2.1% were found to be no longer eligible and were excluded. Seventy-five percent of eligible participants confirmed their BBD biopsy and granted permission to review their pathology slides. In response to our slide requests, we received pathology specimens for 55% of the study subjects. The primary reason given by pathology departments for not sending slides or tissue blocks was that the specimens had been destroyed or were no longer available (33%). There were no significant differences in the success of obtaining slides for breast cancer cases and controls. A detailed description of the steps of specimen collection and exclusions of selected cases and controls during the collection process are included in Table 1. An additional 156 women were excluded for the following reasons: reported bilateral BBD, but tissue specimen did not identify breast side (*n *= 2), report of breast cancer either before study start or after the 1994/1995 follow-up cycle (*n *= 10), and date of benign breast diagnosis was within six months of the breast cancer diagnosis date (cases) or index date (controls) (*n *= 144).

### Pathology review

Slides were coded by a research assistant and submitted to one of two collaborating pathologists (SJS, JLC) in a blinded fashion. The pathologists independently reviewed the BBD biopsy slides. Any slide identified as having either questionable atypia or atypia was jointly reviewed by the two pathologists. For each set of slides reviewed, a detailed work sheet was completed quantifying the extent of proliferative and atypical changes, morphologic features, and other details. BBDs were classified according to the Page classification system [23] into one of three categories: nonproliferative, proliferative without atypia, and atypical hyperplasia. Upon pathology review, it was determined that 29 women with an original BBD diagnosis had evidence of carcinoma *in situ *(*n *= 25) or invasive carcinoma (*n *= 4). These women were excluded from further analysis. There were 353 breast cancer cases and 1,495 controls with pathology slides available from their BBD biopsy.

### Assessment of alcohol consumption

Information on alcohol consumption was obtained from semiquantitative food frequency questionnaires. In NHS I, questions regarding alcohol consumption were asked in 1980, 1984, 1986, and 1990. Women were asked about their average consumption of beer, wine, and liquor separately in the prior year. One drink was considered equal to one can or bottle of beer, a 4-ounce glass of wine, or one drink or shot of liquor. Participants were asked to select from the following categories: almost never, 1 to 3 per month, 1 per week, 2 to 4 per week, 5 to 6 per week, 1 per day, 2 to 3 per day, 4 to 6 per day, 6 or more per day. Similarly, women in NHS II answered questions on consumption of alcohol in the 1989 and 1991 questionnaires. In 1991, the NHS II alcohol questions were expanded to include red wine, white wine, light beer, regular beer, and liquor. Total alcohol consumption per questionnaire cycle was calculated by adding the alcohol contributions from beer, wine, and liquor. Grams of ethanol per day were calculated based on the following equivalents of 12.8 g for regular beer, 11.3 g for light beer, 11.0 g for wine, and 14.0 g for liquor. Alcohol consumption in NHS I has been shown to be valid and highly reproducible in repeated assessments [24].

Women were assigned the alcohol exposure from the cycle preceding the diagnosis of breast cancer. Cases and controls in NHS I with diagnosis (or index) dates preceding the initial food frequency questionnaire administered in 1980 were excluded from the analysis (*n *= 36 cases, 129 controls). If alcohol consumption was missing from the questionnaire before the index date, the exposure from the preceding cycle was assigned. The Spearman correlation between reported alcohol consumption in questionnaire cycles was 0.80 (*P*<0.0001) or greater for all consecutive cycles. Women with missing alcohol exposure data from two cycles preceding the index date were excluded from the analyses (*n *= 35 cases, 143 controls). Thus, there were 282 patients with breast cancer and 1,223 controls eligible for this study with both BBD pathology and detailed alcohol exposure preceding their index date.

### Analytic methods

Odds ratios (ORs) and 95% confidence intervals (CIs) were determined using logistic regression models controlling for matching factors (age, year of BBD biopsy) and follow-up time using the SAS software package (version 8.0; SAS institute, Cary, NC). Follow-up time was defined as the time from BBD diagnosis to breast cancer diagnosis date (cases) or index date (controls). Information on potential confounding variables was obtained from responses on biennial questionnaires. For each 2-year cycle of case–control selection, covariate information was determined from the responses on questionnaires immediately preceding the cycle in which the breast cancer case was diagnosed. In addition to matching factors and follow-up interval, multivariate analyses were adjusted for the following confounders and breast cancer risk factors: first-degree family history of breast cancer (yes/no); quartiles of body mass index (≤ 21.6, 21.7 to 23.6, 23.7 to 26.6, ≥ 26.7 kg/m^2^); age at menarche (<12, 12, 13, ≥ 14 years); age at first birth/parity (nulliparous; one to four children, and age at first birth if ≤ 24 years of age; one to four children, and age at first birth if >24 years of age; five or more children, and age at first birth if ≤ 24 years of age; five or more children age at first birth >24); duration of postmenopausal hormone use (never, <5 years, ≥ 5 years); and menopausal status/type of menopause (premenopausal, natural, bilateral oopherectomy, other or unknown type of menopause).

Analyses examining the combined effect of benign breast histology and alcohol consumption were based on a cross-classified variable that used nonproliferative breast disease and consumption of 0 g of alcohol per day as the reference group. To examine whether the association between alcohol consumption and breast cancer was modified by benign breast histology, we conducted a likelihood ratio test to assess statistical significance of an interaction term using an ordered scale for benign breast histology and alcohol consumption.

## Results

Among the controls selected in this nested case–control study, women with atypical hyperplasia were older at biopsy and had their biopsies slightly later (1983 versus 1979) than women with nonproliferative benign histology (Table 2). Women with atypical hyperplasia had a greater prevalence of family history of breast cancer and were less likely to be premenopausal at benign biopsy than women with nonproliferative breast disease. In addition, recent alcohol consumption was greatest for women with atypical hyperplasia and lowest for women with nonproliferative breast disease (Table 2). This result is unexpected, given that previous analysis in the NHS II cohort indicated that recent alcohol consumption is not associated with an increased risk of proliferative breast disease, including atypical hyperplasia [13]. Adjusting for age did not change the interpretation of the results in Table 2. For the other breast cancer risk factors presented in Table 2, there was no clear pattern of association with benign histology.

Overall, women with proliferative breast disease without atypia had a 50% greater risk of breast cancer than women with nonproliferative disease (OR = 1.54) (Table 3). Women with atypical hyperplasia had a more than fourfold increased risk of breast cancer in comparison with women with nonproliferative breast disease (OR = 4.43) (Table 3).

In this nested case–control study, there was no increased risk of breast cancer among women drinking ≥ 15 g of alcohol per day compared with nondrinkers (OR = 0.86; 95% CI 0.50 to 1.46).

The combined effects of alcohol consumption and category of BBD histology are presented in Table 4. Among women with nonproliferative BBD, recent alcohol consumption was positively associated with breast cancer risk: those consuming ≥ 15 g of alcohol per day had a nonsignificant 67% increased risk of breast cancer (OR = 1.67; 95% CI 0.65 to 4.34).

Among women with nonproliferative BBD, there was a 10% increased risk of breast cancer per 5 g increase in daily alcohol consumption (OR = 1.10; 95% CI 0.95 to 1.27). Among women with proliferative BBD without atypia, there was no increased risk of breast cancer associated with a 5-g increase in daily alcohol consumption (OR = 0.97; 95% CI 0.87 to 1.08). Similarly, among women with proliferative BBD with atypia, there was no evidence that increasing alcohol consumption increases risk: a 5-g increase in alcohol consumption was associated with a nonsignificant 12% reduction in breast cancer risk (OR = 0.88; 95% CI 0.73 to 1.06). The interaction between alcohol consumption and BBD category was not statistically significant (*P *= 0.20).

Analyses limited to invasive breast cancers (*n *= 232 cases) showed similar results. We were limited in power to adequately assess this relation according to menopausal status and estrogen receptor status of the cancer.

## Discussion

To our knowledge, this is the first study to prospectively examine the effects of alcohol with proliferative BBD in relation to subsequent breast cancer risk. The results of this study indicate that recent alcohol consumption does not increase the risk of breast cancer to a greater extent among women with proliferative BBD than in women with nonproliferative BBD.

Previous epidemiologic studies have examined the effect of alcohol consumption among women with a self-report of BBD, with inconsistent results [1,16-18]. In a cohort study conducted in the Netherlands, van den Brandt and colleagues reported a nonsignificant 2.5-fold increased risk of breast cancer among women with BBD who drank ≥ 15 g of alcohol per day compared with nondrinkers (*P *for trend = 0.15) [16]. A pooled analysis of six cohort studies, including the Netherlands cohort just mentioned, reported no significant interaction between alcohol consumption and BBD with respect to breast cancer risk (*P *= 0.23) [1]. In the California Teachers Study, the joint effect of history of BBD and high alcohol consumption was associated with a twofold increased risk of breast cancer (relative risk (RR) = 1.97; 95% CI 1.39 to 2.79), while nondrinkers with biopsy-confirmed BBD had a 35% increased risk of breast cancer (RR = 1.35; 95% CI 1.05 to 1.73) in comparison with nondrinkers with no history of BBD [17].

Benign breast conditions are a heterogeneous group of diseases and therefore may not all respond to alcohol exposure in the same manner. Although all of the women in this study had a biopsy removing their benign lesion, we were operating under the assumption that the BBD is a marker of susceptibility and the remaining breast tissue may have a similar susceptibly to the effects of alcohol later in life. Nonproliferative breast diseases comprise a large proportion of reported BBD biopsies. The inconsistencies observed between our study and previous studies may be due to the heterogeneous nature of BBD and variability in distribution of BBD types between different studies.

One proposed mechanism by which alcohol may influence breast cancer risk is by increasing circulating estradiol levels, as has been observed in controlled feeding studies in both premenopausal and postmenopausal women [6,7]. A second potential mechanism is that alcohol may function as a cocarcinogen, inhibiting detoxification of carcinogens, or by impairing clearance of carcinogens [3,8-10]. There are data suggesting that alcohol may act early in the carcinogenic process [6-9], as well as later, functioning as a tumor promoter [3].

The current study failed to support our *a priori *hypothesis and suggests that recent alcohol consumption does not contribute additional risk to women with proliferative breast disease. One explanation may be that women with proliferative breast diseases are further along on the continuum to breast cancer, and are already at an elevated risk of breast cancer, which is no longer affected by alcohol. In contrast, women with nonproliferative breast disease are not as far along the pathway to breast cancer and specific exposures such as alcohol may exhibit harmful effects and influence the risk of breast cancer.

Byrne and colleagues conducted a similar study in the Nurses' Health Study to examine the effects of postmenopausal hormone use among women with BBD [25]. Similarly, the results were contrary to their *a priori *hypothesis and they observed no elevated risk of breast cancer among women with proliferative breast diseases according to current use of hormones or duration of use, again suggesting that these women may be at such an increased risk of breast cancer that the additional effect of exogenous hormone use is minimal or none.

**Figure 1:**
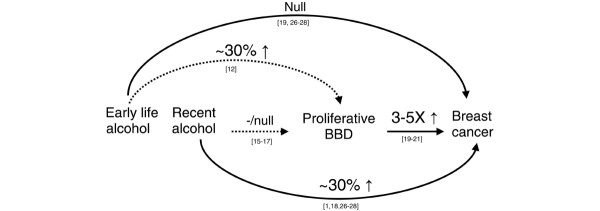
Schematic description of relation between alcohol consumption, proliferative benign breast disease (BBD), and breast cancer. Solid lines indicate well-established associations and dotted lines indicate less-established relations.

In Figure 1, we have schematically described the evidence relating alcohol exposure to breast cancer. It is well accepted that recent alcohol consumption increases the risk of breast cancer [1,16-18,26-28] and there is little evidence that alcohol intake early in life affects breast cancer risk [17,26-28]. The three studies examining alcohol and proliferative breast disease suggest that recent alcohol intake does not increase the risk of proliferative breast disease [13-15] and may in fact be inversely related to it, with estimates ranging from a 10% [13] to a 77% [14] reduction in risk comparing the highest category of alcohol consumption with nondrinkers. These results, along with those from the current study, suggest that the association observed between recent alcohol consumption and breast cancer may not be mediated through proliferative breast disease. However, Byrne and colleagues examined alcohol consumption between the ages of 18 and 22 and proliferative breast disease and reported a 30% increased risk among women consuming ≥ 15 g of alcohol per day compared with nondrinkers (RR = 1.33, 95% CI 1.05 to 1.69) [13], suggesting that only very early alcohol consumption may affect proliferative BBD.

The progression from tumor initiation to breast cancer is not well defined. One hypothesis is that the pathway from normal tissue to breast cancer arises from a series of preinvasive lesions: benign proliferative changes, atypical hyperplasia, and carcinoma *in situ*. If all breast cancers arise from benign lesions, the results of this study, in conjunction with other data, imply that there may be a narrow window of time when alcohol consumption affects breast cancer risk. An alternative explanation may be that there are multiple pathways to breast cancer and pathways involving proliferative benign breast lesions may not be influenced by alcohol consumption. A third possibility is that the magnitude of risk associated with benign proliferative lesions, and in particular atypical hyperplasia, is so much greater than the effect of alcohol that we were underpowered to detect more subtle increases in risk attributable to alcohol in this study.

Although this is one of the largest studies of its kind, the number of women with proliferative breast disease and high alcohol consumption was small. We were underpowered to examine this relation in greater detail with regard to menopausal status and estrogen receptor status of tumors. A potential concern of the study is that the final study population represents 37% of those originally selected. The study protocol required collection of pathologic specimens in order to have unified review of histologic sections. The major limitation of this is that many hospitals routinely destroy specimens after 5 or 10 years. As a result, many potential cases and controls were excluded, which could result in potential selection bias. There were no significant differences in reasons for loss comparing cases to controls, indicating that any differences that do occur are likely to be due to chance.

## Conclusion

In this study, we observed that women with proliferative BBD experienced no additional risk of breast cancer with increased alcohol consumption. It remains unclear whether alcohol acts early, acts late, or has multiple effects on breast cancer risk. Future studies with greater numbers should examine menopausal and hormone receptor status of the cancers in order to provide additional information on both timing of relevant exposure and biologic mechanisms.

## Abbreviations

BBD = benign breast disease; CI = confidence interval; NHS (I, II) = Nurses' Health Study (I, II); OR = odds ratio; RR = relative risk.

## Competing interests

The author(s) declare that they have no competing interests.

## Authors' contributions

RMT conducted the analysis and wrote the manuscript. CB participated in the concept design and data collection. HJB participated in the writing and editing of the manuscript. BR provided statistical support. SJS participated in the concept and design, data collection, and manuscript editing. JLC participated in the concept and design, data collection, and manuscript editing. GAC provided funding for the study and participated in the concept and design, data collection, and manuscript editing. All authors read and approved the final manuscript.

## Figures and Tables

**Table 1:** Cases and controls included in benign breast disease pathologic specimen collection^a^

Cases	Controls	Selection of participants
1,080	4,353	Participants initially selected
-29	-85	Participant died or withdrew from study subsequent to selection
-265	-1,090	We did not pursue collection of specimens, because:
		• participant denied having biopsy or had cyst aspiration only
		• participant could not recall hospital or hospital no longer exists
		• participant denied permission to seek specimens
		• biopsy contained cancer
-379	-1,406	We were unable to obtain requested samples
		• specimens were no longer available or destroyed
		• hospital had no record of the patient or biopsy
		• hospital had no record of the patient or biopsy
-33	-113	Specimens received were not usable for study
		• slides were not of good quality/not evaluable
		• specimens did not contain breast tissue
		• were from wrong person or incorrect date
**374**	**1,659**	**Eligible participants for whom we collected usable specimens**

^a^Nurses' Health Study I (1976 to 1994) and II (1989 to 1995).

**Table 2:** Characteristics of controls in the nested case–control study according to benign breast disease category

	Category of benign breast disease		
	
Descriptive characteristic	Nonproliferative	Proliferative without atypia	Atypical hyperplasia
Mean			
Age at biopsy (y)	42.5	43.7	50.1
Year of biopsy^b^	1979	1980	1983
Age at menarche (y)	12.7	12.5	12.9
Age at first birth^c ^(y)	24.8	24.9	25.0
Age at menopause^d^	46.6	46.5	47.2
Body mass index, kg/m^2^	25.1	24.6	24.5
Parity	2.8	2.7	2.9
Recent alcohol consumption, g/day	4.8	5.4	6.6
Frequency, %			
Premenopausal at biopsy	76.5	76.1	57.1
Family history of breast cancer	11.2	13.3	18.3
Nulliparous	6.5	8.2	5.6
Natural menopause ^d^	46.8	50.7	48.5
Bilateral oophorectomy^d^	20.1	16.8	17.5

^a^Nurses' Health Study I (1976 to 1994) and II (1989 to 1995). ^b^Median. ^c^Among parous women only. ^d^Among postmenopausal women.

**Table 3:** Effect of category of benign breast disease histology on breast cancer risk^a^

Benign breast disease category	Cases No. (%)	Controls No. (%)	OR (95% CI)^b^	OR (95% CI)^c^
Nonproliferative	69(24.5)	474 (38.8)	1.00 (Ref)	1.00 (Ref)
Proliferative without atypia	140 (49.7)	623 (50.9)	1.59 (1.16–2.18)	1.54 (1.12–2.11)
Atypical hyperplasia	73 (25.9)	126 (10.3)	4.54 (3.03–6.78)	4.43 (2.93–6.69)
Total	282	1,223		

^a^Nurses' Health Study I (1976 to 1994) and II (1989 to 1995). ^b^Adjusted for age, year of biopsy, and follow-up interval. ^c^Adjusted for age, year of biopsy, and follow-up interval, age at menarche, body mass index, weight change since age 18, age at first birth/parity, menopausal status/type of menopause, duration of postmenopausal hormone use. CI, confidence interval; OR, odds ratio; Ref, reference group for statistical comparisons.

**Table 4:** Combined effect of alcohol and category of benign breast disease histology on breast cancer risk^a^

				Jointly-classified^b^		Stratified^c>^	
Category of BBD	Recent alcohol consumption (g/day)	Cases	Controls	OR (95% CI)^d^	OR (95% CI)^e^	OR (95% CI)^d^	OR (95% CI)^e^

Nonproliferative							
	0	21	178	1.00 (Ref)	1.00 (Ref)	1.00 (Ref)	1.00 (Ref)
	<5	27	165	1.41 (0.76–2.60)	1.42 (0.77–2.65)	1.34 (0.72–2.50)	1.46 (0.75–2.85)
	5–15	14	95	1.27 (0.62–2.62)	1.25 (0.60–2.60)	1.31 (0.63–2.74)	1.15 (0.53–2.53)
	≥ 15	7	36	1.67 (0.66–4.27)	1.67 (0.65–4.34)	1.85 (0.71–4.84)	1.90 (0.67–5.39)
	*P *for trend^f^					0.13	0.20
Proliferative without atypia							
	0	46	205	2.04 (1.17–3.57)	1.99 (1.13–3.50)	1.00 (Ref)	1.00 (Ref)
	<5	52	235	1.91 (1.11–3.29)	1.82 (1.05–3.16)	0.99 (0.63–1.55)	0.99 (0.62–1.56)
	5–15	34	123	2.43 (1.34–4.41)	2.40 (1.31–4.39)	1.17 (0.70–1.94)	1.14 (0.67–1.91)
	≥ 15	8	60	1.15 (0.48–2.75)	1.09 (0.45–2.62)	0.59 (0.26–1.33)	0.61 (0.26–1.41)
	*P *for trend^f^					0.49	0.58
Proliferative with atypia							
	0	23	40	5.57 (2.77–11.21)	5.58 (2.74–11.38)	1.00 (Ref)	1.00 (Ref)
	<5	30	45	6.65 (3.43–12.90)	6.48 (3.31–12.70)	1.28 (0.62–2.67)	1.29 (0.60–2.78)
	5–15	14	25	5.35 (2.39–12.01)	5.26 (2.30–12.04)	1.03 (0.42–2.51)	1.02 (0.39–2.68)
	≥ 15	6	16	3.65 (1.27–10.47)	3.46 (1.19–10.09)	0.66 (0.22–1.97)	0.59 (0.19–1.89)
	*P *for trend^f^					0.21	0.17

^a^Nurses' Health Study I (1976 to 1994) and II (1989 to 1995). ^b ^Joint classification of BBD category and alcohol consumption where the reference group is women with nonproliferative BBD and 0 alcohol consumption. ^c ^The analysis is stratified by type of BBD where the reference group is women with 0 alcohol consumption within each category of BBD. ^d^Adjusted for age at diagnosis, year of biopsy, and follow-up interval. ^e^Adjusted for age at diagnosis, year of biopsy, and follow-up interval, age at menarche, body mass index quartiles, family history of breast cancer, parity/age at first birth, menopausal status/type of menopause, duration of postmenopausal hormone use. ^f^Test for trend based on Wald test for continuous alcohol consumption. BBD, benign breast disease; CI, confidence interval; OR, odds ratio; Ref, reference group for statistical comparisons.

## References

[B1] Smith-Warner SA, Spiegelman D, Yaun SS, van den Brandt PA, Folsom AR, Goldbohm RA, Graham S, Holmberg L, Howe GR, Marshall JR0 (1998). Alcohol and breast cancer in women: a pooled analysis of cohort studies. JAMA.

[B2] Longnecker MP (1994). Alcoholic beverage consumption in relation to risk of breast cancer: meta-analysis and review. Cancer Causes Control.

[B3] Singletary KW, Gapstur SM (2001). Alcohol and breast cancer: review of epidemiologic and experimental evidence and potential mechanisms. JAMA.

[B4] Longnecker MP, Berlin JA, Orza MJ, Chalmers TC (1988). A meta-analysis of alcohol consumption in relation to risk of breast cancer. JAMA.

[B5] Ellison RC, Zhang Y, McLennan CE, Rothman KJ (2001). Exploring the relation of alcohol consumption to risk of breast cancer. Am J Epidemiol.

[B6] Brisson J, Merletti F, Sadowsky NL, Twaddle JA, Morrison AS, Cole P (1982). Mammographic features of the breast and breast cancer risk. Am J Epidemiol.

[B7] Boyd NF, Lockwood GA, Byng JW, Tritchler DL, Yaffe MJ (1998). Mammographic densities and breast cancer risk. Cancer Epidemiol Biomarkers Prev.

[B8] Boyd NF, Lockwood GA, Martin LJ, Byng JW, Yaffe MJ, Tritchler DL (2001). Mammographic density as a marker of susceptibility to breast cancer: a hypothesis. IARC Sci Publ.

[B9] Atkinson C, Warren R, Bingham SA, Day NE (1999). Mammographic patterns as a predictive biomarker of breast cancer risk: effect of tamoxifen. Cancer Epidemiol Biomarkers Prev.

[B10] Boyd NF, Connelly P, Byng J, Yaffe M, Draper H, Little L, Jones D, Martin LJ, Lockwood G, Tritchler D (1995). Plasma lipids, lipoproteins, and mammographic densities. Cancer Epidemiol Biomarkers Prev.

[B11] Vachon CM, Kuni CC, Anderson K, Anderson VE, Sellers TA (2000). Association of mammographically defined percent breast density with epidemiologic risk factors for breast cancer (United States). Cancer Causes Control.

[B12] Vachon CM, Kushi LH, Cerhan JR, Kuni CC, Sellers TA (2000). Association of diet and mammographic breast density in the Minnesota breast cancer family cohort. Cancer Epidemiol Biomarkers Prev.

[B13] Byrne C, Webb PM, Jacobs TW, Peiro G, Schnitt SJ, Connolly JL, Willett WC, Colditz GA (2002). Alcohol consumption and incidence of benign breast disease. Cancer Epidemiol Biomarkers Prev.

[B14] Rohan TE, Jain M, Miller AB (1998). Alcohol consumption and risk of benign proliferative epithelial disorders of the breast: a case-cohort study. Public Health Nutr.

[B15] Rohan TE, Cook MG (1989). Alcohol consumption and risk of benign proliferative epithelial disorders of the breast in women. Int J Cancer.

[B16] van den Brandt PA, Goldbohm RA, van 't Veer P (1995). Alcohol and breast cancer: results from The Netherlands Cohort Study. Am J Epidemiol.

[B17] Horn-Ross PL, Canchola AJ, West DW, Stewart SL, Bernstein L, Deapen D, Pinder R, Ross RK, Anton-Culver H, Peel D (2004). Patterns of alcohol consumption and breast cancer risk in the California Teachers Study cohort. Cancer Epidemiol Biomarkers Prev.

[B18] Willett WC, Stampfer MJ, Colditz GA, Rosner BA, Hennekens CH, Speizer FE (1987). Moderate alcohol consumption and the risk of breast cancer. N Engl J Med.

[B19] Page DL, Dupont WD, Rogers LW, Rados MS (1985). Atypical hyperplastic lesion of the female breast. A long-term follow-up study. Cancer.

[B20] London ST, Connolly JL, Schnitt SJ, Colditz GA (1992). A prospective study of benign breast disease and the risk of breast cancer. JAMA Med Assoc.

[B21] Carter CL, Corle DK, Micozzi MS, Schatzkin A, Taylor PR (1988). A prospective study of the development of breast cancer in 16,692 women with benign breast disease. Am J Epidemiol.

[B22] Colditz GA (1990). The Nurses' Health Study: findings during 10 years of follow-up of a cohort of U.S. women. Curr Probl Obstet Gynecol Fertil.

[B23] Dupont WD, Page DL (1985). Risk factors for breast cancer in women with proliferative breast disease. N Engl J Med.

[B24] Giovannucci E, Colditz G, Stampfer MJ, Rimm EB, Litin L, Sampson L, Willett WC (1991). The assessment of alcohol consumption by a simple self-administered questionnaire. Am J Epidemiol.

[B25] Byrne C, Connolly JL, Colditz GA, Schnitt SJ (2000). Biopsy confirmed benign breast disease, postmenopausal use of exogenous female hormones, and breast carcinoma risk. Cancer.

[B26] Holmberg L, Baron JA, Byers T, Wolk A, Ohlander EM, Zack M, Adami HO (1995). Alcohol intake and breast cancer risk: effect of exposure from 15 years of age. Cancer Epidemiol Biomarkers Prev.

[B27] Freudenheim JL, Marshall JR, Graham S, Laughlin R, Vena JE, Swanson M, Ambrosone C, Nemoto T (1995). Lifetime alcohol consumption and risk of breast cancer. Nutr Cancer.

[B28] Tjonneland A, Christensen J, Thomsen BL, Olsen A, Stripp C, Overvad K, Olsen JH (2004). Lifetime alcohol consumption and postmenopausal breast cancer rate in Denmark: a prospective cohort study. J Nutr.

